# Caries of Jaw

**Published:** 1881-02

**Authors:** Geo. G. Gale

**Affiliations:** Quebec, Canada


### CARIES OF THE JAW.

BY GEO. G. GALE, A.M., M.D., QUEBEC, CANADA.

Miss B——, Æt. 20. Seven months ago disease began in the left side of the inferior maxilla. The gum of the diseased side is swollen, there are two fistulous openings at the inferior border and to the inside, discharging thin, offensive, bloody matter. In Royle's "Materia Medica" (allopathic) one of the effects of phosphorus is to produce carries of the lower jaw. Because "*Similia Similibus Curantur*" is the law of cure. *Phosphorus* in the C.M. potency cured her in six weeks.

